# Involvement of the cerebellum in migraine

**DOI:** 10.3389/fnsys.2022.984406

**Published:** 2022-10-13

**Authors:** Mengya Wang, Joseph O. Tutt, Nicholas O. Dorricott, Krystal L. Parker, Andrew F. Russo, Levi P. Sowers

**Affiliations:** ^1^Department of Neuroscience and Pharmacology, University of Iowa, Iowa City, IA, United States; ^2^Department of Biology and Biochemistry, University of Bath, Bath, United Kingdom; ^3^Department of Psychiatry, University of Iowa, Iowa City, IA, United States; ^4^Department of Molecular Physiology and Biophysics, University of Iowa, Iowa City, IA, United States; ^5^Department of Neurology, University of Iowa, Iowa City, IA, United States; ^6^Center for the Prevention and Treatment of Visual Loss, Veterans Administration Health Center, Iowa City, IA, United States; ^7^Department of Pediatrics, University of Iowa, Iowa City, IA, United States

**Keywords:** migraine, cerebellum, pain, CGRP, sensory processing

## Abstract

Migraine is a disabling neurological disease characterized by moderate or severe headaches and accompanied by sensory abnormalities, e.g., photophobia, allodynia, and vertigo. It affects approximately 15% of people worldwide. Despite advancements in current migraine therapeutics, mechanisms underlying migraine remain elusive. Within the central nervous system, studies have hinted that the cerebellum may play an important sensory integrative role in migraine. More specifically, the cerebellum has been proposed to modulate pain processing, and imaging studies have revealed cerebellar alterations in migraine patients. This review aims to summarize the clinical and preclinical studies that link the cerebellum to migraine. We will first discuss cerebellar roles in pain modulation, including cerebellar neuronal connections with pain-related brain regions. Next, we will review cerebellar symptoms and cerebellar imaging data in migraine patients. Lastly, we will highlight the possible roles of the neuropeptide calcitonin gene-related peptide (CGRP) in migraine symptoms, including preclinical cerebellar studies in animal models of migraine.

## Introduction

Migraine is a debilitating primary headache disorder commonly characterized by moderate or severe headaches that can be aggravated by routine activity (ICHD-3, 2018). Migraine is often accompanied by sensory abnormalities, such as photophobia, phonophobia, nausea, vertigo, and allodynia. The disorder affects 14.4% of the global population, partitioned into 18.9% of females and 9.8% of males (Stovner et al., 2018). Headache disorders, including migraine, are the second leading global cause of disability (GBD 2017 Disease and Injury Incidence and Prevalence Collaborators, 2018; Stovner et al., 2018). Migraine types include those with and without aura, which can be broadly grouped as episodic or chronic, that is less or greater than 15 headache days a month, respectively. Episodic migraine can evolve into chronic migraine. It was believed that migraine patients improperly filter sensory inputs (e.g., somatosensory, visual, and auditory inputs, which results in migraine symptoms (e.g., head pain, photophobia, and phonophobia, respectively).

The cerebellum is widely known for integrating non-motor sensory signals and controlling motor function (Manto et al., 2012). There is, however, a growing realization indicating cerebellar involvement in perceptual (Baumann et al., 2015), emotional (Adamaszek et al., 2017), and cognitive functions (Koziol et al., 2014; Van Overwalle et al., 2020). Of note, there is mounting evidence to support cerebellar involvement in pain and motor response to pain (Moulton et al., 2010), which are both phenotypes exhibited by migraine patients. In addition to the implication in pain phenotypes, the cerebellum communicates to pain/migraine-related regions; is implicated in the other migraine symptoms; and, more importantly, is altered in the migraine patient imaging studies (Vincent and Hadjikhani, 2007; Kros et al., 2018). The objective of this review is to summarize and update the clinical and preclinical studies that link the cerebellum to migraine. Cerebellar symptoms in migraine patients and a comprehensive summary of imaging studies on the cerebellum in migraine patients with/without exposure to sensory stimuli will be reviewed. The possibility that cerebellar CGRP actions may contribute to migraine will be discussed, along with cerebellar findings in migraine animal models, which may provide insight into migraine pathophysiology.

## The Current Therapeutics for Migraine

There are two migraine management goals: acute treatments to relieve attacks or preventive treatments to reduce attack frequency and severity. There are non-specific anti-migraine medications for acute and preventative treatment, e.g., non-steroidal anti-inflammatory drugs (NSAIDs) and antiemetics for abortion, and beta blockers and anticonvulsants for prevention (Mayans and Walling, 2018; Ha and Gonzalez, 2019). There are also specific anti-migraine therapies, including triptans (5-HT_1B/1D_ receptor agonists), ditans (5-HT_1F_ receptor agonists), gepants [calcitonin gene-related peptide (CGRP) receptor antagonists] as abortive drugs, and CGRP and CGRP receptor monoclonal antibodies as preventative treatments (Eigenbrodt et al., 2021). Rimegepant and atogepant, belonging to gepants, were recently approved for migraine prevention (Tao et al., 2022) and eptinezumab for acute use in the emergency department (Benemei et al., 2022). Mechanisms underlying migraine prevention or acute treatment following symptom onset are not clear. However, it has been suggested that CGRP actions contribute to both abortive and prophylaxis management in that CGRP-based drugs can achieve both management goals. Among these anti-migraine drugs, 30%–40% of migraine patients do not respond to triptan (Lombard et al., 2020), 50% do not respond to ditans (Mecklenburg et al., 2020), 50% do not respond to CGRP-based drugs (Edvinsson et al., 2018), and more than 50% do not respond to the non-specific anti-migraine preventative treatments (Deen et al., 2017). Further understanding of migraine pathophysiology is necessary to develop new and more effective therapeutics.

Migraine pathogenesis is multifactorial. The failure of efficacy in the non-responders to current medications suggests that CGRP is not the only cause of migraine and that other neuropeptides are involved; e.g., pituitary adenylate cyclase-activating peptide (PACAP-38) works independently of CGRP or 5-HT_1B/1D/1F_ receptors (Kuburas et al., 2021; Ernstsen et al., 2022). In addition, CGRP-based drugs act on the periphery with low penetration across the blood-brain barrier (Edvinsson and Warfvinge, 2019); eg., a preclinical study reported that galcanezumab, one of the FDA-approved CGRP antibodies targeting the CGRP peptide, demonstrated limited blood-brain barrier penetration when injected peripherally into rats (Johnson et al., 2019). Penetration into the cerebellum was 0.18% of the plasma concentration, and penetration into the hypothalamus, the spinal cord, or the prefrontal cortex was ~0.3% of the plasma concentration (Johnson et al., 2019). The lack of central penetration may be another reason that some migraine patients do not benefit from current therapeutics. This emphasizes that the central mechanisms underlying migraine pathogenesis should be considered. While central mechanisms contribute to migraine pathophysiology, there is still a significant dearth of understanding about the neuroanatomical correlates in migraine. Central neural circuits controlling sensory perception may play a critical role in this disease state. The cerebellum represents a prime sensory integration center in the brain that may govern some of these pathological phenotypes in migraine.

## Cerebellar Anatomy Controlling Motor Processing

The cerebellum has three main divisions: the cerebrocerebellum, the vestibulocerebellum, and the spinocerebellum (Purves et al., 2018). It is widely accepted that the cerebellum plays a role in motor function (Manto et al., 2012). The cerebrocerebellum, occupies most of the lateral cerebellar hemisphere and projects primarily to the lateral cerebellar nucleus (also known as the dentate nucleus). The cerebrocerebellum receives signals from cerebral cortical areas and sends signals back to the same areas, and in this manner, engages in motor planning (Purves et al., 2018). The vestibulocerebellum, comprising the caudal lobes of the cerebellum, participates in balance control and vestibuloocular regulation. The spinocerebellum contains the median and paramedian zones of the cerebellar hemispheres. Specifically, the paramedian (also called intermediate) zone, which projects primarily to the interposed nucleus, is involved in distal muscle movements, while the median zone (also called the vermis), which projects to the medial cerebellar nucleus (MN, also known as the fastigial nucleus), is involved in movements of axial and proximal muscles (Purves et al., 2018). In addition, the oculomotor vermis (lobules VI and VII) and the MN control eye movement (Manto et al., 2012).

For motor performance, the cerebellum receives dynamic sensory information and subsequently corrects or optimizes movements *via* outputs to different regions (Jueptner and Weiller, 1998). Specifically, the cerebellum receives vestibular, visual, tactile, or proprioceptive sensory information from the spinal cord, vestibular, trigeminal, and dorsal column nuclei *via* mossy fibers, as well as from the inferior olive *via* climbing fibers (Rondi-Reig et al., 2014); e.g., the vestibulocerebellum receives signals from and sends signals to the vestibular complex to control eye and head movements (Purves et al., 2018). The interposed nucleus receives signals from the red nucleus *via* the inferior olive and signals back to the red nucleus (Siegel and Sapru, 2019; Basile et al., 2021). The red nucleus is a region involved in motor control and nociceptive processing in the brainstem (Basile et al., 2021). The MN receives inputs from and sends projections to the reticular and vestibular complex to modulate the lower motor neurons (Purves et al., 2018; Siegel and Sapru, 2019). It is likely that these circuits are shared between motor functions and sensory processing suggesting that they not only lay a foundation for movement control but also have implications for altered sensory processing in migraine.

## Cerebellar Role in Pain Processing

In the previous section, the likely role of the cerebellum in sensory processing is mentioned. Considering that pain is an unpleasant sensory and emotional experience associated with actual or potential tissue damage (Sandkuhler, 2009), several studies have investigated the potential link between the cerebellum and pain processing.

### Cerebellar circuitry in sensory and emotional dimensions of pain

The sensory dimension of pain in the body is mainly mediated by the spinothalamic tract, which is composed of the spinal cord to the ventroposterolateral thalamus (Kandel et al., 2021). In the head, it is primarily mediated by the trigeminothalamic tract comprising the trigeminal ganglion to the spinal trigeminal nucleus (SpV) and then to the ventroposteromedial thalamus (Kandel et al., 2021) which is a crucial circuit involved in migraine headache attacks (Burstein et al., 2000; Noseda et al., 2019). The ventroposterolateral and ventroposterormedial thalamus are both posterior thalamic nuclei. In addition, the intralaminar thalamus (including the parafasciculus and centromedian thalamus), the amygdala, and the PAG are all involved in the emotional dimension of pain (Ab Aziz and Ahmad, 2006; Siegel and Sapru, 2019). Indeed, sensitization of the SpV and connected third-order trigeminovascular thalamic neurons contributes to the development of cephalic allodynia and migraine headache (Landy et al., 2004; Burstein et al., 2015; Dodick, 2018).

**Figure 1:**
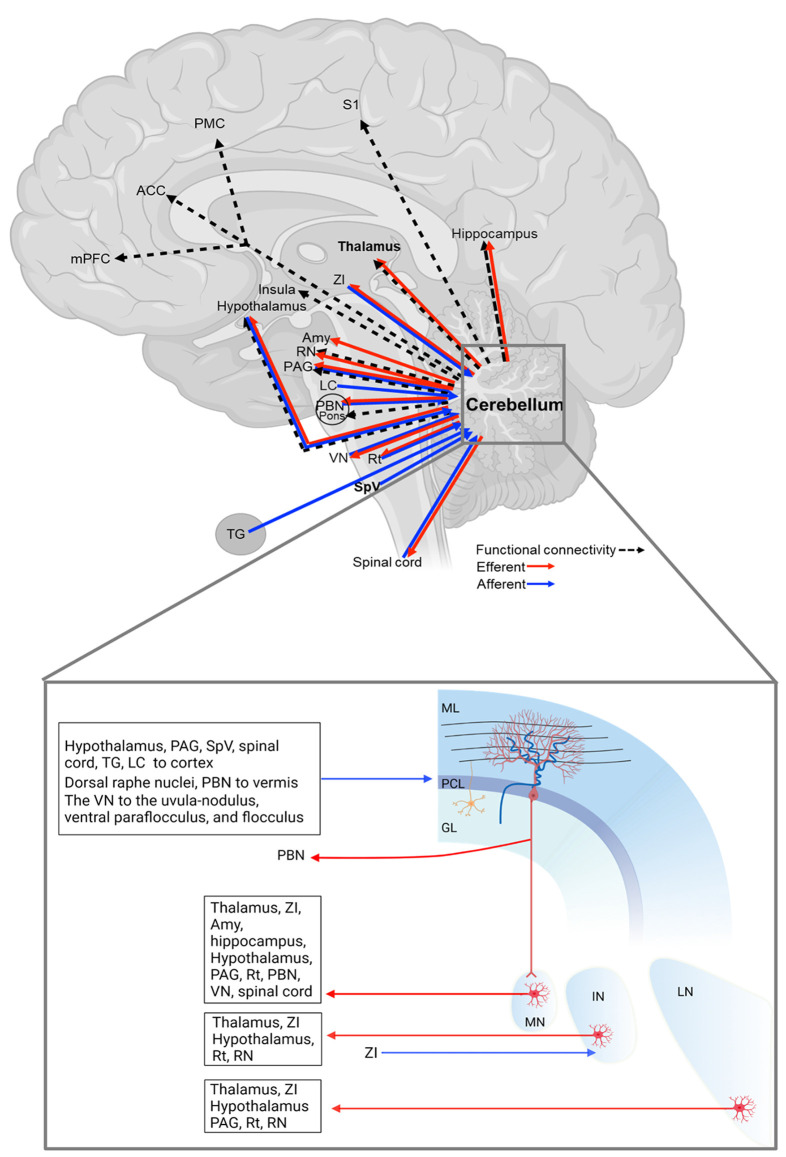
Cerebellar connections with pain/migraine-relevant brain regions. The details of each connection are in Table 1. GL, granular cell layer; IN, interposed cerebellar nucleus; LN, lateral cerebellar nucleus; ML, molecular layer; MN, medial cerebellar nucleus; PCL, Purkinje cell layer. The figure was created with BioRender.com.

In this section, we will discuss that the cerebellum is structurally connected to regions involved in processing sensory and emotional aspects of pain (Figure 1 and Table 1). Here we organized the cerebellar connections with other regions into sensory and emotional circuits. While the functional relevance of these connections is, at present, poorly defined, their existence suggests a cerebellar influence on these regions towards behavior relevant to migraine.

**Table 1:** Cerebellar connections with pain or migraine-relevant brain regions.

**Connections**	**Type**	**Details**	**Ref.**
**Cerebellum-Primary somatosensory cortex (S1)**	FC	In migraine patients compared to HCs in the interictal phase • ↓ FC between the right S1 and the right cerebellum posterior lobe (lobule VIIIb)	Zhang et al. (2017)
**Cerebellum-Premotor cortex (PMC)**	FC	In migraine patients compared to HCs during the interictal phase • ↓ FC between the right dorsal premotor cortex and ipsilateral cerebellar lobule VIII	Qin et al. (2020)
**Cerebellum- ACC**	FC	Mice receiving IS at low and high frequency; Mice receiving IS at low frequency and NTG • ↑ cerebellar FCs with the ACC	Jia et al. (2019)
**Cerebellum-Insula**	FC	Mice receiving IS at low and high frequency; Mice receiving IS at low frequency and NTG • ↑ cerebellar FCs with the insula	Jia et al. (2020)

		In migraine patients compared to HCs • ↑ FC between the right posterior insula and the bilateral cerebellum	Ke et al. (2020)
**Cerebellum-Medial prefrontal cortex (mPFC)**	FC	In migraine patients compared to HCs • ↓ FC between the left Crus I and the default mode network components (including mPFC)	Ke et al. (2020)

		In migraine patients compared to HCs • ↑ FC between the right cerebellum and the right mPFC.	Jin et al. (2013)
**Cerebellum-Thalamus**	Circuitry →	• The MN → ventromedial, ventrolateral, centrolateral, mediodorsal, parafascicular, suprageniculate and posterior thalamic nuclei	Teune et al. (2000) and Fujita et al. (2020)
		• The interposed nucleus → the ventrolateral and ventroposterior thalamic nuclei	Teune et al. (2000) and Baldacara et al. (2008)
		• The lateral cerebellar nucleus → the thalamus	Teune et al. (2000) and Baldacara et al. (2008)

	FC	In migraine patients compared to HCs during the interictal phase. • ↑ FC between the left lateral geniculate nucleus and the ipsilateral cerebellum	Zhang et al. (2020)

	FC	In migraine patients receiving trigeminal stimuli compared to HCs • ↓ FC between Left Crus I (ipsilateral to the stimulation) and the left thalamus and some cortical areas	Mehnert and May (2019)

		Episodic migraine patients administered NTG orally compared to their own baseline • FC change between the right thalamus and the cerebellum during the prodromal and full-blown phase	Martinelli et al. (2021)
**Cerebellum- zona incerta (ZI)**	Circuitry → ←	• The MN → ZI	Fujita et al. (2020)

		• The interposed nucleus ↔ (reciprocally) the ZI	Teune et al. (2000) and Ossowska (2020)

		• The lateral nucleus → ZI	Teune et al. (2000)
**Cerebellum-Amygdala (Amy)**	Circuitry →	• The MN directly → the amygdala (histological degeneration studies)	Heath and Harper (1974)
**Cerebellum-Hippocampus**	Circuitry →	• The MN directly → the hippocampus (histological degeneration studies)	Heath and Harper (1974)

	FC	In migraine patients compared to HCs during the interictal phase • ↑ FC between the hippocampus and the cerebellum	Wei et al. (2020)
**Cerebellum-Hypothalamus**	Circuitry →	• Three deep cerebellar nuclei → the hypothalamus contralaterally	Haines and Dietrichs (1984)

		• The MN sends GABAergic fibers → the hypothalamus	Cao et al. (2013)

	←	• The hypothalamus → the cerebellar cortex bilaterally with the ipsilateral preponderance	Dietrichs (1984) and Haines and Dietrichs (1984)
	FC	In migraine patients compared to HCs during the interictal phase • ↑ FC between the hypothalamus, and cerebellar Crus I&II and lobules V&VI	Moulton et al. (2014)
**Cerebellum-periaqueductal gray (PAG)**	Circuitry →	• The MN and lateral nucleus →PAG	Teune et al. (2000); Frontera et al. (2020); and Fujita et al. (2020)

	←	• The PAG → the cerebellar cortex	Dietrichs (1983)

	FC	In migraine with ictal allodynia compared to migraine without ictal allodynia • ↑ FC between PAG and the cerebellum	Schwedt et al. (2014b)
**Cerebellum-Reticular formation (Rt)**	Circuitry →	• The three deep nuclei → the Rt	Teune et al. (2000) and Fujita et al. (2020)

	←	• Dorsal raphe nuclei → the cerebellar vermis	Dietrichs (1985)

		• The Rt → the developing cerebellum *via* serotonergic fibers	Bishop et al. (1988)
∣rule
**Cerebellum-Red nucleus (RN)**	Circuitry →	• The interposed and lateral nucleus →the RN	Siegel and Sapru (2019) and Basile et al. (2021)

	FC	In migraine patients compared to HCs during the interictal phase • ↑FC between the right red nucleus and the ipsilateral cerebellum	Huang et al. (2019)
**Cerebellum-parabrachial nucleus (PBN)**	Circuitry → ←	• The MN and a small number of Purkinje cells in the anterior cerebellar vermis → the PBN	Supple and Kapp (1994), Teune et al. (2000), and Fujita et al. (2020)

		• The PBN sends multilayered fibers →the anterior cerebellar vermis	Supple and Kapp (1994)

	FC	In migraine patients administered NTG to induce a headache, compared to their own baseline. • ↑FC between the pons and the cerebellar tonsils	Karsan et al. (2020)
**Cerebellum-Locus coeruleus (LC)**	Circuitry ←	• The LC → the cerebellar cortex	Dietrichs (1985), Dietrichs (1988)
**Cerebellum-Vestibular nucleus (VN)**	Circuitry →	• The paraflocculus, the flocculus, and the uvula-nodulus → the VN	Tabata et al. (2002) and Barmack (2016)

	←	• The VN → the uvula-nodulus, ventral paraflocculus, and flocculus	Barmack (2016)

	→	• The MN → the VN	Bagnall et al. (2009) and Fujita et al. (2020)
**Cerebellum-Spinal trigeminal nucleus (SpV)**	Circuitry ←	• The SpV and principle sensory nucleus → the cerebellar vermal, medial, and lateral zones	Ikeda (1979), Hayashi et al. (1984), Ohya et al. (1993), and Ge et al. (2014)

		• These trigemino-cerebellar projection neurons predominantly express the vesicular-glutamate transporter 1 (VGLUT1)	Ge et al. (2014)
**Cerebellum-Trigeminal ganglion (TG)**	Circuitry ←	• The TG → ipsilaterally in Crus I and II, the paramedian lobule, the lateral cerebellar nucleus, and each lobe of the parafloccular cortex	Jacquin et al. (1982)
**Cerebellum-spinal cord**	Circuitry →	• The MN →the spinal cord	Fujita et al. (2020)

	←	• Spinal cord regions, including the central cervical nucleus, the dorsal nucleus, the lumbar and sacral precerebellar nuclei, lumbar border precerebellar cells, and from dispersed neurons of the deep dorsal horn and laminae 6–8 → the cerebellar cortex, mainly the vermis	Sengul et al. (2015)

HCs, healthy controls; FC, functional connectivity; ACC, the anterior cingulate cortex; IS, inflammatory soup; NTG, nitroglycerin; →←, projecting direction; ↑, increase; ↓, decrease.

### Sensory-related connections

#### Spinal cord and brainstem

The cerebellar cortex—predominantly the vermis—is innervated by multiple spinal cord regions, including the contralateral central cervical nucleus, the ipsilateral dorsal nucleus, the lumbar and sacral precerebellar nuclei, lumbar border precerebellar cells, and dispersed neurons of the deep dorsal horn and laminae 6–8 (Sengul et al., 2015), and the MN sends contralateral projections to the spinal cord (Fujita et al., 2020; Figure 1 and Table 1), highlighting the reciprocal connections of the cerebellum and spinal cord.

Another important region in the ascending pain pathway is the SpV, which receives sensory inputs from the cranial meninges and extracephalic skin and relays these inputs to pain processing regions, the thalamic nuclei (Burstein et al., 2010). Studies also demonstrated that the SpV sends projections to the cerebellar cortex (Ikeda, 1979; Hayashi et al., 1984; Ohya et al., 1993; Ge et al., 2014; Figure 1 and Table 1). These SpV-cerebellar neurons predominantly express the vesicular-glutamate transporter 1 (Ge et al., 2014). It is possible that the cerebellum could be acting to process trigeminal inputs from the SpVefferents and acting as a putative nociceptive processing center (Ruscheweyh et al., 2014). Moreover, an earlier study showed that the primary trigeminal afferents from the trigeminal ganglion terminate in cerebellum regions, including ipsilateral Crus I and II, the paramedian lobule, the lateral cerebellar nucleus, and the paraflocculus (Jacquin et al., 1982; Figure 1 and Table 1). The inputs from the trigeminal sensory neurons highlight this cerebellar connection for a potential role in the processing of sensory input during migraine attacks.

The parabrachial nucleus (PBN) is a region known to modulate multiple aversive behaviors (Palmiter, 2018). The MN sends projections to the PBN (Supple and Kapp, 1994; Teune et al., 2000; Fujita et al., 2020), which relays signals to the cerebellar vermis (Dietrichs, 1985; Supple and Kapp, 1994; Figure 1 and Table 1). Finally, the cerebellum is connected with reticular formation (Bishop et al., 1988; Teune et al., 2000; Fujita et al., 2020), including the dorsal raphe nuclei (Dietrichs, 1985) for pain modulation (Wang and Nakai, 1994), and the red nucleus for motor control and nociceptive processing (Huang et al., 2019; Siegel and Sapru, 2019; Basile et al., 2021; Figure 1 and Table 1). Together, the vast structural connectivity of the cerebellum with pain processing regions implies a cerebellar hand in sensory evaluation, and dysregulation of these pathways could contribute to the multitude of sensory abnormalities observed during the prodrome and headache phases of migraine.

#### Thalamus and subthalamus

As mentioned above, the thalamus is the key component in pain modulation, and cerebellar innervation of the thalamic region could contribute to it (Figure 1 and Table 1). The lateral cerebellar nucleus sends projections to the thalamus (Teune et al., 2000; Baldacara et al., 2008). The interposed nucleus innervates the ventrolateral and ventroposterior thalamic nuclei (Teune et al., 2000; Baldacara et al., 2008). The ventroposterior region receives input from ascending trigeminal sensory neurons (Graziano et al., 2008). The MN projects to various thalamic nuclei, including ventromedial, ventrolateral, centrolateral, mediodorsal, parafascicular, suprageniculate thalamic nuclei, and the nucleus in the posterior thalamus (PoT; Teune et al., 2000; Fujita et al., 2020). The PoT integrates signals from the SpV and retinal ganglion cells (Noseda et al., 2010) and is thought to be involved in light-aversive behavior, acutely relevant to migraine (Sowers et al., 2020). Together, cerebellar innervation of the thalamus may contribute to migraine, e.g., thalamic sensitization during migraine.

Moreover, the deep cerebellar nuclei are connected to the zona incerta (ZI), adjacent to the thalamus (Teune et al., 2000; Fujita et al., 2020; Ossowska, 2020). The ZI exerts inhibitory control on all higher-order thalamic nuclei, including the PoT *via* GABAergic projections (Bartho et al., 2002). The ZI acts to gate peripheral inputs into the PoT, depending on the behavioral state (Trageser and Keller, 2004). Interestingly, disinhibition of the trigeminal ZI-PoT- primary somatosensory cortex circuit has been observed in models of chronic pain (Masri et al., 2009) and could be anticipated to accompany migraine (Brennan and Pietrobon, 2018).

### Emotion-related regions

The experience of pain contains an emotional-affective dimension, including anxiety and fear. The cerebellum is connected to regions involved in emotional processing (Figure 1 and Table 1) and is thought to be involved in the neural circuitry driving anxiety (Otsuka et al., 2016). The thalamus serves as a hub that regulates both sensory and affective aspects of pain, whose connections to the cerebellum was discussed in section “Sensory-related connections”.

The cerebellum is also known to be reciprocally connected to the periaqueductal gray (PAG; Dietrichs, 1983; Teune et al., 2000; Frontera et al., 2020; Fujita et al., 2020), an antinociceptive processing center (Basbaum and Fields, 1978; Morgan et al., 1997). Specifically, the MN and the lateral nucleus project to the PAG (Teune et al., 2000; Frontera et al., 2020; Fujita et al., 2020), and the PAG projects to the cerebellar cortex, including the anterior lobe, the simple lobule, crus I, crus II, the paramedian lobule and the posterior vermis (Dietrichs, 1983). A direct or indirect circuit from the ventrolateral PAG, which modulates fear responses to imminent threats (Vianna and Brandao, 2003; Wright et al., 2019), to the lateral vermal lobule VIII is thought to be necessary for fear-evoked freezing behavior (Koutsikou et al., 2014).

The locus coeruleus, implicated in stress and panic, sends projections to the vermal (Dietrichs, 1985, 1988), intermediate and lateral zones of the cerebellar cortex, with abundant projections in the vermal and intermediate zones (Dietrichs, 1988). In addition, the MN is connected to the hippocampus, the amygdala (Heath and Harper, 1974) implicated in a role in emotional processing (Zhang et al., 2016), and the hypothalamus perhaps related to the development of the autonomic components of the migraine attack (Dietrichs, 1984; Haines and Dietrichs, 1984; Cao et al., 2013). Notedly, even though Heath and Harper reported monosynaptic cerebellar inputs to the hippocampus and amygdala (Heath and Harper, 1974), two recent studies doubted this observation (Watson et al., 2019; Fujita et al., 2020) but the physiological connections are well studied. Furthermore, several psychiatric disorders are comorbid with migraine, including anxiety and depression (Merikangas and Stevens, 1997; Balaban et al., 2011; Dresler et al., 2019). Given that cerebellar gray matter volume and activity are altered in anxiety patients (Tillfors et al., 2002; Warwick et al., 2008; Bing et al., 2013; Talati et al., 2015; Ke et al., 2016; Wang T. et al., 2016; cerebellar structural abnormalities in migraine patients are detailed in section “Cerebellar volume”) and that the cerebellum is structurally connected to multiple affective processing centers, it is feasible that the cerebellum modulates affective aspects of migraine attacks. Dissecting the function of cerebellar circuits in preclinical models is warranted and may provide novel insights for future targeted stimulation-based migraine therapeutics.

## Cerebellar impairment alters pain-related symptoms in humans

Pain evaluation was performed in patients with cerebellar infarction. Compared to healthy controls, patients with infarction limited to the cerebellum displayed hyperalgesia to thermal and repeated mechanical stimuli, which were applied to the forearm (Ruscheweyh et al., 2014). The heat hyperalgesia in the ipsilateral side of the infarction site was more prominent (Ruscheweyh et al., 2014). Offset analgesia is a form of endogenous pain inhibition characterized by disproportionately large reductions in pain intensity ratings evoked by small decreases in stimulus intensity (Yelle et al., 2009; Oudejans et al., 2015). In patients with cerebellar infarction in both anterior and posterior areas, offset analgesia was reduced, suggesting deficient descending pain inhibition in patients with cerebellar infarction (Ruscheweyh et al., 2014). In addition, children with resection extending into Crus I/II showed decreased cold pain tolerance compared to healthy controls (Silva et al., 2019). These two studies suggest that cerebellar impairment could lead to hyperalgesia.

## Cerebellar manipulation modulates pain in animals

### Pain-related behaviors

The presence of cerebellar anatomical connections to pain-related brain regions and observations that cerebellar impairment alters pain in humans suggest a possible cerebellar involvement in pain. However, the causal or modulatory relationship between the cerebellum and pain has yet to be discerned. Preclinical studies provide a valuable avenue to reveal the cerebellar mechanisms underlying pain. While it is impossible to confirm that animals are experiencing pain, methods that assess “pain-like” behaviors have been developed. In general, these methods are divided into two types based on whether the stimulus is applied, e.g., thermal, cold, or mechanical stimulus (Deuis et al., 2017). Widely used stimulus-evoked methods include von Frey, hot plate, and tail-flick tests (Deuis et al., 2017). The non-stimulus, or spontaneous, methods include grimace and burrowing tests (Deuis et al., 2017).

### Cutaneous nociception

Approximately 60% of migraine patients reported cutaneous allodynia (Lipton et al., 2008), and here we discuss the evidence linking the cerebellum and cutaneous nociception. Early studies conducted by Russell (1894) and Sprague and Chambers (1959) revealed that cerebellar destruction altered responses to sensory stimuli in animals. Later, the relationship was further explored in awake squirrel monkeys with electrical stimulation of the cerebellar regions in response to tail shock (Siegel and Wepsic, 1974). An increase in nociceptive thresholds was found by electrically stimulating the posterior vermal lobules VI (lobulus simplex) and VII-IX; the anterior intermediate lobules IV-V (culmen); the rostral lateral cerebellar-interpossitus nuclear-brachium conjunctivum in intermediate lobe (Siegel and Wepsic, 1974), suggesting that stimulation of these areas is antinociceptive. Among these regions, the anterior intermediate lobules IV-V and rostral lateral cerebellar-interpossitus nuclear-brachium conjunctivum in the intermediate lobe displayed dramatic analgesia at stimulation of 0.2 mA (Siegel and Wepsic, 1974). However, when the current was increased to 0.8 mA, stimulation of the bilateral lobules HVIII (the paramedian lobes) decreased the tail shock nociceptive threshold. Altogether, this study suggests a regional specificity of the analgesic effect, which is also dependent upon the stimulation current.

Consistent with this finding, Dey and Ray (1982) later found that electrical stimulation at the culmen or at centralis but close to the culmen region of the anterior cerebellum induced post-stimulation analgesia lasting ~5–10 min in rats at the intensity of 0.06–0.3 mA (Dey and Ray, 1982). Moreover, administration of morphine into the culmen region of the anterior cerebellum evoked analgesia in rats. This analgesic effect could be blunted by intraperitoneal (i.p.) injection of naloxone, a non-selective opioid receptor antagonist. Ablation of culmen and centralis, shortened analgesia duration after i.p. morphine while lobulus simplex and declive lesion had a trend to prolong i.p. morphine-induced analgesia (Dey and Ray, 1982). These two studies (Siegel and Wepsic, 1974; Dey and Ray, 1982) show a similar result when the culmen is targeted, suggesting that the culmen region is critical in regulating nociception, probably *via* connections with the brainstem reticular formation. Thus, one can speculate that the cerebellum, specifically the culmen, exerts its antinociceptive action by activating the brainstem pain suppression mechanism (Dey and Ray, 1982). In contrast, Hardy found that stimulation of the cerebellar cortex did not induce thermal nociceptive response (Hardy, 1985). However, the exact region of the cerebellar cortex was not mentioned.

### Visceral reflex

The role of the cerebellum in the visceral nociceptive reflex was also studied. Injection of DL-homocysteic acid, an NMDA receptor agonist, into the MN decreased the elicited abdominal reflex (Saab and Willis, 2002). Congruently, the injection of bicuculline, a GABA-A receptor antagonist, into the MN also decreased the reflex (Saab and Willis, 2002). Administration of bicuculline is believed to inhibit Purkinje cells, consequently releasing the MN from its inhibitory influence (Saab and Willis, 2002). In addition, when DL-homocysteic acid was injected into the cerebellar vermal lobule VIII, the reflex was induced, while no effect was observed from DL-homocysteic acid injection into the lateral cerebellar nucleus (Saab and Willis, 2002). This study suggests that the vermal lobule VIII and the MN produced pronociceptive and antinociceptive effects, respectively, in response to the visceral noxious stimulation.

Later, another study demonstrated that administration of glutamate into the MN of rats with chronic visceral hypersensitivity increased the pain threshold, and decreased amplitude and abdominal withdrawal reflex scores (Zhen et al., 2018). This phenotype could be abolished by delivering 3-MPA (a glutamate decarboxylase inhibitor) into the MN, suggesting that glutamate stimulation of the MN exerts a protective action on chronic visceral hypersensitivity (Zhen et al., 2018).

### Anxiety

In addition, cerebellar manipulation modulated anxiety. One study using a mutant mouse line with Purkinje cell death reported that mutants spent more time in the open arms of the elevated plus maze, suggesting an anti-anxiety phenotype (Hilber et al., 2004). Bilateral lesion of the MN in juvenile rats was shown to alter anxiety in adulthood although it was not clear whether the anxiety was enhanced or decreased (Helgers et al., 2020), accompanied by enhancement of local field coherence between the medial prefrontal cortex and the sensorimotor cortex (Helgers et al., 2020) and epigenetic dysregulation of the GABAergic system in the nucleus accumbens and the oxytocin system in the prefrontal cortex (Helgers et al., 2021). Cerebellar manipulation can also change fear responses, an affective component of pain. For more details, please refer to Sacchetti et al. (2005).

## Neuronal activities in pain-related brain regions

### Thalamus

Cerebellar stimulation may manipulate neuronal activities of other regions, including the parafasciculus thalamus, the habenula and the spinal cord, which could contribute to pain. At the circuit level, sciatic nerve stimulation in rats changed the neuronal firing rate in the parafasciculus thalamus, which could be changed by lateral cerebellar nucleus stimulation in an intensity-dependent manner (Liu et al., 1993). For instance, 0.2 mA inhibited most of the nociceptive-on cells (the cells showing an increase in firing rate following the noxious stimulation) in the parafasciculus thalamus, while 0.4 mA activated these neurons (Liu et al., 1993). Considering that the MN and lateral cerebellar nuclei have direct connections to the parafasciculus thalamus (Teune et al., 2000; Fujita et al., 2020), the cerebellum may modulate nociception *via* the parafasciculus thalamus. In addition, cerebellar electrical stimulation also increased the nociceptive-on neuronal responses to tail pinch in the habenula, while transcranial electrostimulation, lateral hypothalamic electrical stimulation, and dorsal raphe electrical stimulation showed opposite effects on nociceptive-on cells in the habenula (Dong et al., 1992).

### Spinal cord

The spinal cord is a critical region for regulating pain and headaches. Electrical (0.1–0.15 mA) or DL-homocysteic acid stimulations of the posterior cerebellar vermal lobule VI modulated the neuronal activity of the lumbosacral spinal cord in response to non-noxious and noxious visceral (colorectal distention) or somatic (brush, pressure, and pinch) stimuli: an increase in spinal cord neuronal activity in response to visceral stimuli was observed after cerebellar stimulation, while varied responses to somatic stimuli were seen in rats (Saab et al., 2001). It is possible that the posterior cerebellar vermis exerts its pronociceptive action *via* the inhibition of deep cerebellar nuclei, therefore suppressing the descending pain inhibition pathway.

In contrast, stimulation of the left intermediate hemisphere of the anterior cerebellar cortex significantly decreased the activity of spinal cord dorsal horn neurons bilaterally in response to mechanical stimuli, suggesting the anterior cerebellum may exert an antinociceptive action *via* activating the descending inhibition pathway (Hagains et al., 2011). Altogether, stimulation of the posterior and anterior cerebellum produced opposite effects on spinal cord neuronal activity (Saab et al., 2001; Hagains et al., 2011), and both studies agree that the cerebellum impacts nociception at least in part through the descending pain inhibition pathway.

Furthermore, DL-homocysteic acid injection into the MN increased neuronal responses in dorsal column nuclei to somatic non-noxious stimuli in rats (Saab et al., 2002). The increased neuronal responses in dorsal column nuclei by MN stimulation may be a result of impacting the dorsal column–medial lemniscus pathway directly or the descending pathway indirectly. It should be noted that opposite effects of the MN to pain responses were found after applying innocuous somatic stimuli (increasing activity in dorsal column nuclei indicative of increasing pain; Saab et al., 2002) and noxious visceral stimuli (decreasing visceral nociceptive reflex indicative of decreasing pain; Saab and Willis, 2002) after stimulation of the MN in a comparable paradigm. This seemingly contradictory finding suggests further exploration is necessary to explain the role of the cerebellum in processing non-noxious and noxious information.

## Human Studies on The Cerebellum and Migraine

Migraine is characterized by pulsating and moderate or severe pain on the unilateral side of the head. Migraine patients also reported cutaneous allodynia (pain in response to a non-nociceptive stimulus; Schwedt, 2013; ICHD-3, 2018), and other sensory and motor abnormalities, such as visual, auditory, olfactory, and vestibular hypersensitivity (Schwedt, 2013) with photophobia being the most bothersome symptom other than pain during migraine attacks (Munjal et al., 2020). The cerebellar hand in sensory and pain processing implies a cerebellar influence on head pain, cutaneous allodynia, and sensory abnormalities experienced by migraine patients. In this section, we will talk about the cerebellar symptoms including motor and non-motor dysfunction, imaging studies about cerebellar alterations in migraine patients, and cerebellar manipulations in migraine patients.

### Cerebellar symptoms in migraine patients

The cerebellum coordinates both motor and non-motor functions. This section will discuss motor (e.g., motor coordination, vestibular, and oculomotor functions) and non-motor dysfunction in migraine patients.

### Motor dysfunction

As mentioned above, vestibular nuclei and the cerebellum are closely connected and work in concert to influence posture, equilibrium, and vestibuloocular eye movements (Purves et al., 2018). Vestibular motor dysfunction was exhibited in migraine patients from the general population. Stabilometric assessment of migraine patients revealed increased body sway relative to healthy controls during both ictal (Anagnostou et al., 2019) and interictal periods (Ishizaki et al., 2002; Anagnostou et al., 2019). However, another study reported no difference in body sway between migraine patients and non-migraine controls (Koppen et al., 2017). Reasons for disparate results are unknown; however, the authors suggest that different sample sizes, patients selected from different cohorts, and a blind design might change the results (Ishizaki et al., 2002; Koppen et al., 2017; Anagnostou et al., 2019). All the same, it was surprising that 8.5% of migraine patients in the second study had ischemic cerebellar lesions located in the posterior lobe (Koppen et al., 2017). These lesions apparently affected fine motor skills but not body sway or other non-motor cerebellar functions (Koppen et al., 2017). Similarly, vestibular symptoms were often predisposed by ischemic or inflammatory lesioning of the cerebellum or brainstem (Kim et al., 2005; Brandt and Dieterich, 2017). Furthermore, a recent study revealed that migraine patients exhibit increased postural sway relative to non-headache controls across a range of light intensities (Pinheiro et al., 2020). This interaction between visual light sensitivity and the corresponding imbalance phenotype suggests a link between the visual system and motor processing in the cerebellum (Pinheiro et al., 2020), but the mechanisms are unclear.

Migraine patients experiencing moderate or severe vestibular symptoms may fall into the diagnostic criteria of vestibular migraine, a subtype of migraine. Vestibular symptoms include positional vertigo, visually-induced vertigo, head motion-induced dizziness with nausea, etc. (ICHD-3, 2018). Approximately 10%–30% of patients in headache and dizziness clinics are diagnosed with vestibular migraine, with the condition affecting about 1%–3% of the total population (Neuhauser et al., 2006; Formeister et al., 2018; Wattiez et al., 2020) and accounting for 10% among migraine patients (ICHD-3, 2018). The current treatments for acute vestibular migraine attacks include triptans (which are effective in 40% of patients) and antiemetic medications (Shen et al., 2020). Prophylactic treatments include selective calcium channel blockers (which reduced vertigo and headaches in ~65% of patients) and the antiepileptic drug, topiramate (which was effective among 80% of patients; Shen et al., 2020). CGRP-based drugs can improve both migraine and vestibular symptoms in 18 out of 25 patients suffering from vestibular migraine (Hoskin and Fife, 2022). Given the role that the cerebellum and vestibular nuclei play in motor function, targeting the cerebellum may improve the vestibular impairments.

Central ocular motor disorders are a common co-morbidity seen in individuals with vestibular migraine (Neugebauer et al., 2013). This neurological condition is characterized by nystagmus and saccades (Neugebauer et al., 2013). In migraine patients, studies have shown deficiencies in nystagmus and saccadic accuracy, indicative of defective oculomotor function (Harno et al., 2003). The oculomotor vermis is related to saccades and pursuit initiation, and the vestibulocerebellum modulates the vestibularocular reflex (Kheradmand and Zee, 2011; Lal and Truong, 2019). Collectively, these findings suggest that cerebellar deficiencies may partly account for the faulty oculomotor processing displayed by migraine patients.

Motor dysfunction was also exhibited in a subtype of migraine patients, familial hemiplegic migraine (FHM). FHM is an autosomal dominant subtype of migraine with aura characterized by fully reversible motor weakness (ICHD-3, 2018), and displays cerebellar ataxia and nystagmus (Thomsen et al., 2002; Dichgans et al., 2005).

### Non-motor dysfunction

Non-motor function in migraine patients may contribute to attack-related disability and interfere with work performance and personal life. However, it is often neglected by clinicians and little is known regarding cognitive dysfunction in migraine patients (Gil-Gouveia and Martins, 2019). Individuals with FHM1, a type of FHM [FHM type will be discussed in detail in Section “Cerebellar changes in familial hemiplegic migraine (FHM)”], were subjected to a series of validated assessment procedures, testing for a range of cerebellar phenotypes (e.g., fine motor skills, vestibular motor function measured by body sway, visuospatial ability, and learning-dependent timing; Koppen et al., 2017). Visuospatial ability and learning-dependent timing require cognitive mechanisms, including working memory, attention, and planning. The cerebrocerebellum is believed to participate in cognitive function (Koppen et al., 2017). Results showed that besides fine motor and vestibular motor dysfunction, FHM1 patients demonstrated defective cerebellar performance in parameters for non-motor function tests (Koppen et al., 2017).

The experience of time is the foundation for information processing and motor behavior, the impairment of which can influence an individual’s life (Zhang et al., 2012). Time perception and estimation involve cognitive functions, e.g., perception, attention, and memory (Zhang et al., 2012). A cerebellar role in the performance of timing tasks is well established (Parker, 2015; Ohmae et al., 2017; Tanaka et al., 2021). Migraine patients demonstrated impaired timing estimation in the milliseconds’ range (Zhang et al., 2012).

Together, these observations suggest that the cerebellum may have an expansive role in migraine symptomology that extends beyond mere motor output. Future studies will be required to determine whether the specific subregions of the cerebellum and its respective connections are distinctively associated with a specific migraine symptom.

## Cerebellar alterations in migraine patients: imaging studies

There is an abundance of imaging studies identifying structural, activity, and functional cerebellar changes in migraine patients. These reports have allowed a glimpse into the cerebellar role in migraine pathophysiology.

### Cerebellar structural changes without sensory stimuli application in migraine patients

#### Cerebellar volume

One interesting finding from imaging studies is the change in cerebellar volume observed in migraine patients. Given that migraine attacks are intermittent, the modification of cerebellar morphology might occur over time. Cerebellar atrophy was observed, and a negative correlation was identified between the migraine disease duration and cerebellar volume (Demir et al., 2016). The majority of the cerebellum is comprised of gray matter, inclusive of the cerebellar cortex and deep cerebellar nuclei. Cerebellar gray matter volume (GMV) decreases were observed in migraine patients, detected during the interictal phase (Jin et al., 2013; Yang et al., 2018; Bonanno et al., 2020; Chou et al., 2021). In contrast, some studies reported an increase in GMV in some cerebellar regions in migraine patients. For example, Mehnert and May demonstrated a GMV increase in lobules VI, VIIb, VIIIa, Crus I, and Crus II in the right cerebellum in migraine patients compared to healthy controls (Mehnert and May, 2019). Furthermore, the GMV decrease in the right VI lobule was correlated with higher attack frequency, and the GMV decrease in the right lobule V was correlated with the disease duration (Mehnert and May, 2019). Another group selectively included patients with high-frequency migraine (10–30 headache days/month). These patients with poor outcomes (<50% reduction in baseline headache days or frequency increase over 2 years) displayed greater GMV in the right Crus II and left Crus I in the cerebellum than healthy controls, and in the bilateral VIIIa and left Crus I in the cerebellum than patients with good outcomes (≥50% reduction over 2 years) during the interictal phase (Liu H. Y. et al., 2020). There was a correlation between the disease duration and GMV in the right cerebellar VIIIa, and between 2-year headache frequencies and GMV in the bilateral VIIIa (Liu H. Y. et al., 2020). This seemingly contrasting data might be attributed to the states when migraine patients were scanned, and the population of migraine patients included in the studies.

In addition to gray matter, the microstructure of white matter is abnormal in migraine patients. Specifically, diffusion tensor imaging (DTI) reveals that the comparison to healthy controls, migraine patients have decreased axonal integrity in vermal lobule VI extending to the bilateral lobules V and VI. These analyses also revealed myelin damage in the right inferior cerebellum peduncle which is composed of cerebellar inputs and outputs (Tae et al., 2018; Qin et al., 2019).

In summary, studies investigating cerebellar structural alterations in migraine patients are not consistent. Longitudinal studies with larger sample sizes and consideration of disease progression would increase the scope for more in-depth analysis moving forward.

#### Cerebellar activity

Neuronal activity of the cerebellum has been extensively investigated in different migraine conditions with positron emission tomography (PET) and functional magnetic resonance imaging (fMRI) scans. During the interictal phase, regions in the bilateral posterior lobe in the cerebellum showed spontaneous activity deficiencies in migraine patients compared to healthy controls (Wang J.-J et al., 2016). The value of spontaneous activity in the left superior cerebellum could discriminate migraine patients from healthy controls, and the value positively correlated with the baseline headache intensity (Yin et al., 2020). When comparing migraine patients with aura to those without aura, the activity amplitude in the bilateral cerebellum was higher in the aura group than in those without aura during the interictal phase (Farago et al., 2017). Importantly, when the migraine phase was taken into consideration, cerebellar activation was observed in female migraine patients in the ictal phase compared to the interictal phase (Afridi et al., 2005). In addition, vestibular migraine patients displayed dramatically increased metabolism in the bilateral cerebellum in the ictal phase compared to the interictal phase (Shin et al., 2014). Vestibular rehabilitation, which aimed to alleviate vestibular symptoms, enhanced the spontaneous activity of the left posterior cerebellum (Liu L. et al., 2020). It suggests that left cerebellar hyperactivity might compensate for the deficits in the vestibular system (Liu L. et al., 2020) and that targeting the cerebellum may be a potential avenue to improving vestibular symptoms in migraine patients. It should be noted that there is no observed consistency in the activation of the specific cerebellar regions between reports. Despite this caveat, studies demonstrated that migraine induces cerebellar activation relative to healthy controls, which is phase-dependent.

#### Cerebellar functional connectivity

In addition to changes in cerebellar structure and activity in migraine patients, functional connectivity changes are observed (Figure 1 and Table 1). Understanding these functional connectivity changes between the cerebellum and other brain regions will be critical in understanding migraine pathophysiology.

Compared to healthy controls during the interictal phase, functional connectivity increases are observed (Figure 1 and Table 1) between the left lateral geniculate nucleus (the relay center for the visual pathway located in the posterior thalamus) and the ipsilateral cerebellum (Zhang et al., 2020). In addition, functional connectivity was increased in a number of studies in the following locations: between the right red nucleus and the ipsilateral cerebellum (Huang et al., 2019); between the hippocampus and the cerebellum (Wei et al., 2020); between the hypothalamus and cerebellar Crus I and II and lobules V and VI (Moulton et al., 2014); between the right posterior insula and the bilateral cerebellum (Ke et al., 2020); and between the right medial prefrontal cortex and the ipsilateral cerebellum (Jin et al., 2013).

One study found decreased functional connectivity between left Crus I and the default mode network components, including the medial prefrontal cortex in migraine patients compared to healthy controls (Ke et al., 2020). The default mode network has been linked to cognitive and social processing (Li et al., 2014). Functional connectivity between the left Crus I and the left medial prefrontal cortex negatively correlated with migraine frequency (Ke et al., 2020). Du group reported that decreased functional connectivity (Figure 1 and Table 1) was observed between the primary somatosensory cortex and the ipsilateral cerebellar lobule VIIIb (Zhang et al., 2017), and between the right dorsal premotor cortex and the ipsilateral cerebellar lobule VIII in migraine patients compared to healthy controls during the interictal phase (Qin et al., 2020). However, conflicting data was observed as that Qin et al. did not observe changes in the functional connectivity between the primary somatosensory cortex and the cerebellum (Qin et al., 2020). The reason is not clear.

Different ratios of male to female migraine patients or varied migraine phenotypic profiles can affect functional connectivity results. For example, functional connectivity between the PAG and the cerebellum is higher in migraine with ictal allodynia than without ictal allodynia (Schwedt et al., 2014b). These data paint a complex picture of migraine-related functional connectivity and suggest more preclinical studies are necessary to precisely define how specific cerebellar circuits contribute to migraine.

#### Cerebellar infarcts

Intriguingly, one study showed that migraine patients had a higher risk of subclinical infarcts in the cerebellar posterior circulation territory, and this risk increased with higher attack frequency (Kruit et al., 2004). Notably, there was no significant difference in infarcts in other locations (anterior/carotid circulation, basal ganglia, and corona radiata/centrum semiovale) in migraine patients compared to control subjects (Kruit et al., 2004). Later the same group observed that the cerebellar silent infarcts were always in the posterior lobe in the cerebellar hemispheres and paramedian region (Koppen et al., 2017). The mechanism of infarction remains to be elucidated. Further studies investigating how silent cerebellar infarcts are induced in migraine are necessary, both to further our understanding of migraine pathophysiology and to provide preventive actions for migraine patients with a higher risk of cerebellar infarcts.

### Cerebellar changes with sensory stimuli application in migraine patients

Hyperexcitablity in the brain has been reported *via* electroencephalography (EEG) and fMRI techniques which may contribute to hypersensitivity to various sensory modalities like visual, auditory, olfactory, and somatosensory stimuli in patients who experience migraine (Main et al., 1997; Demarquay et al., 2006; Aurora and Wilkinson, 2007; Ashkenazi et al., 2009). Imaging studies of cerebellar activity and functional connectivity were conducted in migraine patients in response to different stimuli, e.g., visual (Kreczmanski et al., 2019), thermal (Moulton et al., 2011; Maleki et al., 2012, 2021; Russo et al., 2012; Schwedt et al., 2014a), olfactory (Stankewitz and May, 2011), and trigeminal nociceptive (Mehnert and May, 2019) stimuli.

### Visual stimuli

Depending on which visual stimulus was used, flickering or static checkerboards activated different cerebellar regions in migraine patients compared to the rest status (Kreczmanski et al., 2019). Moreover, the flickering checkerboard experiment showed higher left cerebellum activity in migraine patients with aura than those without aura, while the static checkerboard experiment showed greater activity in the right cerebellum (Kreczmanski et al., 2019).

### Thermal stimuli

The cerebellum was activated in both migraine patients and healthy controls upon reception of painful thermal stimuli to the forearm (Schwedt et al., 2014a) or the face (Moulton et al., 2011; Russo et al., 2012) in the interictal phase. However, the cerebellar regions activated by thermal stimulation on the face between migraine patients and healthy controls were different (Moulton et al., 2011; Russo et al., 2012). Differences in cerebellar region activation were also observed in the interictal and ictal phases when responding to a thermal stimulus applied to the hand (Maleki et al., 2021). Additionally, cerebellar functional connectivity with the temporal pole and the entorhinal cortex was increased in response to thermal stimulation of the forehead in migraine patients compared to healthy controls in the interictal phase (Moulton et al., 2011). Interestingly, female migraine patients showed higher activation in the cerebellum and higher deactivation of cerebellar functional connectivity with the insula than males with noxious heat (Maleki et al., 2012). Altogether, these studies suggest that the cerebellum plays a role in processing visual and painful thermal information in migraine patients.

### Olfactory stimuli

May and colleagues observed cerebellar changes during olfactory stimulation in healthy controls and migraine patients. They found that the cerebellum was activated in both healthy controls (Stankewitz et al., 2010) and migraine patients (Stankewitz and May, 2011) who showed higher cerebellar activation in the ictal phase compared to the interictal phase in response to odors (Stankewitz and May, 2011).

### Trigeminal stimuli

May group applied trigeminal stimuli to healthy controls (Stankewitz et al., 2010; Mehnert et al., 2017) and migraine patients (Mehnert and May, 2019). In healthy controls, activation was found in the left cerebellar regions (lobules V, VI, VIIIa and Crus I) and the vermal lobule VIIIa, ipsilaterally to the stimulation site. In contrast, less activation was found in the contralateral right cerebellar hemisphere (lobules I–VI; Mehnert et al., 2017). The left SpV showed higher functional connectivity with the left lobules I–IV. The left lobules VI and VIIIa, and vermal lobule VIIIa showed higher functional connectivity with the thalamus or cortical areas (Mehnert et al., 2017). Later, the same stimulation conditions were applied to migraine patients (Mehnert and May, 2019) as in Mehnert et al. (2017). Compared to healthy controls, left Crus I (ipsilateral to the stimulation) of the migraine patient’s cerebellum showed increased activity and decreased functional connectivity with the left thalamus and some cortical areas in response to trigeminal nociceptive stimulation (Mehnert and May, 2019; Figure 1 and Table 1). Based on the understanding that the cerebellum is indicated to have an inhibitory role in nociception even though there is uncertainty (discussed in Section “Cerebellar role in pain processing”), it can be speculated that the increase of cerebellar activity is to compensate for the dysfunctional cerebellar functional connectivity to cortical areas in migraine patients (Mehnert and May, 2019). Strikingly, treatment with erenumab, a monoclonal antibody targeting the CGRP receptor, reduced cerebellar activation on both sides in migraine patients in response to trigeminal nociceptive stimuli compared to before erenumab treatment (Ziegeler et al., 2020). This finding indicates that erenumab can have central effects, although these are likely secondary to the peripheral effects, and implies that CGRP may contribute to the cerebellar activity abnormalities observed in migraine. These data highlight the complex functional connectivity of the cerebellum in migraine patients.

### Chemical stimuli

In addition to sensory triggers, a peptide trigger, pituitary adenylate cyclase-activating polypeptide-38 (PACAP38), which was reported to induce migraine-like headaches in migraine patients (Schytz et al., 2009; Ghanizada et al., 2020), decreased right cerebellar functional connectivity with default mode network in the early phase of migraine attacks (Amin et al., 2016). Migraine patients administered the migraine trigger nitroglycerin (NTG) can also display a variety of symptoms that closely mirror a migraine attack (Sances et al., 2004). Following NTG administration to migraine patients, the cerebellum showed functional connectivity changes with the right thalamus during the prodromal and full-blown phase (Martinelli et al., 2021), and increased functional connectivity with the pons during the headache phase (Karsan et al., 2020) compared to pre-treatment baseline. These studies suggest that abnormal cerebellar functional connectivity might contribute to the lack of nociceptive modulation in PACAP38- (Amin et al., 2016) and possibly NTG-induced migraine attacks.

### Cerebellar changes in familial hemiplegic migraine (FHM)

FHM represents a small portion of migraine patients which may provide insight into migraine as a more general disease through the study of genes contributing to their migraine phenotypes. It can be divided into three subtypes: FHM1, 2, and 3. FHM1 is caused by mutations in the CACNA1A gene, which encodes the α1A subunit of Cav2.1 (P/Q-type) calcium channel. Cav2.1 channel is vital for neurotransmitter release (Catterall, 1998) and is expressed in the brain—cerebellar Purkinje cells in particular (Westenbroek et al., 1995). These mutations usually lead to enhanced glutamatergic neurotransmission (Sutherland et al., 2019). FHM2 is caused by mutations in ATP1A2, which encodes the α2 subunit of a Na^+^/K^+^ ATPase pump. FHM3 is caused by SCN1A mutations encoding the α1 subunit of the neuronal sodium channel Na_v_1.1.

Cerebellar atrophy was observed in FHM 1 patients (Vighetto et al., 1988; Joutel et al., 1993; Haan et al., 1994; Elliott et al., 1996; Terwindt et al., 1998; Dichgans et al., 2005; Kono et al., 2014). Atrophy was present in the vermis (Vighetto et al., 1988; Joutel et al., 1993; Elliott et al., 1996; Kono et al., 2014), particularly in the anterior vermis (Vighetto et al., 1988; Elliott et al., 1996). Moreover, studies on one FHM patient (Neligan et al., 1977) or subjects with a family relation to FHM patients (Kors et al., 2001; Takahashi et al., 2005) revealed a possible degenerative mechanism, including Purkinje cell death and abnormal dendritic and axonal morphology. These changes were more apparent in the vermis than in the cerebellar hemisphere (Kors et al., 2001). Deep cerebellar nuclei were relatively intact (Kors et al., 2001). However, the other two studies observed not only changes in Purkinje cells but also gliosis in lateral cerebellar nuclei (Neligan et al., 1977; Takahashi et al., 2005). Together, these observations imply that cerebellar degeneration is involved in FHM. It will be interesting to study cerebellar structural changes in FHM mouse models to further understand the mechanisms of the cerebellum in migraine.

Energy metabolism was also investigated in FHM patients. Compared to healthy controls, FHM1 patients showed a significant reduction in *N*-acetyl aspartate and glutamate, as well as an increase in myo-inositol in the superior cerebellar vermis (Dichgans et al., 2005). These findings indicate impairments in neuronal integrity and glutamatergic neurotransmission, and abnormal proliferation of glial cells, respectively. In addition, *N*-acetyl aspartate in the superior cerebellar vermis was significantly correlated with gait ataxia score (Dichgans et al., 2005). Similarly, another study found that FHM1 and FHM2 patients showed a significant decrease in *N-acetyl* aspartate in the cerebellum compared to healthy controls, with the lowest value in FHM1 patients in the interictal phase (Zielman et al., 2014).

These discoveries point to the importance of the metabolism and neuronal functions of the vermis and Purkinje cells in the FHM cerebellar symptoms. N-acetyl aspartate levels might be an early biomarker for neuronal abnormality or disease progression in FHM (Dichgans et al., 2005; Zielman et al., 2014).

### Cerebellar modulations in migraine patients

Studies report that transcranial direct current stimulation of the cerebellum modulated pain intensity in healthy controls (Bocci et al., 2015, 2017). Further, Brighina et al. (2009) applied transcranial magnetic stimulation to migraine patients, with a conditioning stimulus on the right cerebellar cortex and a test stimulus on the left motor cortex. They reported that migraine patients showed a deficit in cerebellar inhibition of the motor cortex compared to healthy controls, which may contribute to the deficit of cortical inhibitory circuits reported in migraine patients (Brighina et al., 2009). Ho et al. (2010) interpreted the cerebellar deficits observed in this study (Brighina et al., 2009) to be a contributor to improper sensory filtering, which is processed in the cortex as painful stimuli. Future studies are necessary to precisely define the mechanisms underlying cerebellar inhibition of cerebral cortical circuits in migraine patients.

## The Cerebellum Contains CGRP and Abundant CGRP Binding Sites

Given the findings described above, it is reasonable to speculate that the cerebellum plays a role in migraine, although whether this is in a causal or merely a regulatory capacity remains to be seen. The underlying mechanisms are unknown. This section discusses the potential contribution of cerebellar CGRP to migraine pathophysiology.

CGRP, a multifunctional neuropeptide, is known to modulate nociception and assist in the onset of migraine. In migraine patients, CGRP levels are elevated in the plasma, cerebrospinal fluid, and saliva, and the infusion of CGRP alone is sufficient to induce a migraine-like headache in 70% of migraine patients (Goadsby et al., 1990; Goadsby and Edvinsson, 1993; Lassen et al., 2002; Juhasz et al., 2005; Petersen et al., 2005; Cady et al., 2009; van Dongen et al., 2017; Russo, 2019). Most notably, CGRP receptor antagonist drugs that block the ligand-receptor binding interaction, are approved by the FDA for the treatment of migraine with efficacy in approximately 50% of sufferers (Olesen et al., 2004; Ho et al., 2008; Connor et al., 2009; Dodick et al., 2018; Reuter, 2018; Skljarevski et al., 2018; Stauffer et al., 2018). Peripheral release of CGRP is suspected to elicit vasodilation and stimulate mast cell degranulation and inflammation in the dural meninges, leading to sustained activation of meningeal nociceptors and causing the prolonged activation of trigeminal primary afferents (Vecchia and Pietrobon, 2012; Russo, 2015, 2019; Iyengar et al., 2017, 2019; Charles, 2018; Messlinger, 2018). This has been proposed to sensitize trigeminovascular neurons in the thalamus leading to central sensitization that primes the brain for a migraine attack (Vecchia and Pietrobon, 2012; Russo, 2015, 2019; Iyengar et al., 2017, 2019; Charles, 2018). Central sensitization is seemingly also induced by the central release of CGRP, which enhances glutamatergic signaling, increases neuronal excitability, and facilitates synaptic transmission (Han et al., 2005; Seybold, 2009; Russo, 2015). However, the precise sites of CGRP action in the central nervous system that contribute to migraine attacks remain to be fully elucidated. Recent studies have presented the posterior thalamus (Sowers et al., 2020) and the cerebellum (Wang et al., 2022a, b) as candidate sites for CGRP action in migraine pathophysiology.

### CGRP expression in the cerebellum

**Figure 2:**
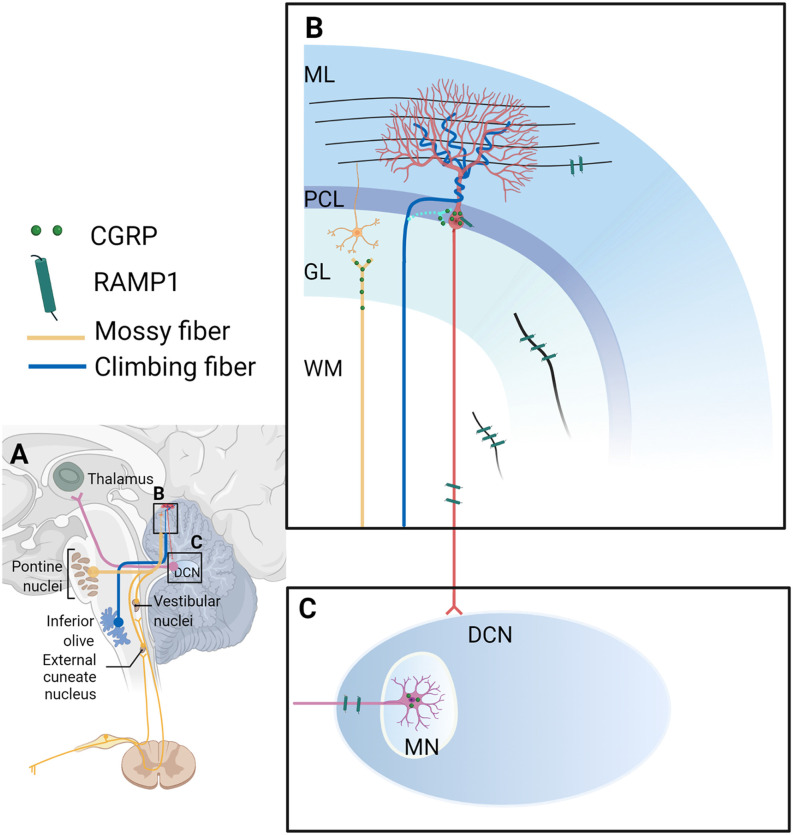
Calcitonin gene-related peptide (CGRP)-positive input to the cerebellum and CGRP and RAMP1 expression in the cerebellum. **(A)** Overview of CGRP-positive inputs to the cerebellar cortex from the inferior olive *via* the climbing fiber (blue line), and the pons, vestibular nuclei, and external cuneate nucleus *via* climbing fibers (yellow line). The deep cerebellar nucleus projects to the thalamus (pink line). Magnified and detailed views of box B and box C are displayed in **(B,C)**, respectively. **(B)** Climbing fibers: During postnatal development, CGRP-positive climbing fiber terminals from the inferior olive converge on the cell somas of nearby Purkinje cells (light blue line; these synapses between climbing fibers and Purkinje cell bodies disappear but synaptic contacts between climbing fibers and Purkinje cell dendrites are established in adulthood (dark blue line; Hashimoto and Kano, 2005; Hashimoto et al., 2009). **Mossy fibers**: In adult brains, CGRP-positive mossy fibers were shown to originate from the brainstem precerebellar nuclei (lateral reticular nucleus, external cuneate nucleus, inferior vestibular nucleus, and basilar pons; yellow line). **Purkinje cells**: In rats, CGRP is distributed in the cytoplasm of the Purkinje cell bodies intracellularly as grains, not found in the dendrites/axons nor other cells (red lines and cell). **RAMP1**: In the rat cerebellum, RAMP1 was found on the surface of the Purkinje cell bodies and their processes, and discovered as fibers in the molecular layer, granular layer (the black line in the GL), and white matter (the black line in the WM). Yellow cell: the granular cell; red cell: the Purkinje cell. **(C)** In the MN of the rat brain, CGRP is localized in the cell somas of large neurons as grains. RAMP1 was also found as processes, but not in the cell somas in the MN. It is unclear where CGRP-expressing neurons in the MN project. CGRP, calcitonin gene-related peptide; DCN, deep cerebellar nucleus; GL, granular cell layer; ML, molecular layer; MN, medial cerebellar nucleus; MW, white matter; PCL, Purkinje cell layer; RAMP1, receptor activity-modifying protein 1. The figure was created with BioRender.com.

An early study by Kawai et al. (1985) highlighted the presence of CGRP in Purkinje cells in rats. Later, studies updated the distribution of CGRP in the cytoplasm of Purkinje cell bodies as grains and not in dendrites, axons, nor other cells in rats (Edvinsson et al., 2011; Warfvinge and Edvinsson, 2019; Warfvinge et al., 2019). In the rat MN, CGRP is localized in the cell somas of large neurons as grains (Warfvinge and Edvinsson, 2019; Figure 2); however, in primates, CGRP is distributed in the cytoplasm of cell bodies and dendrites of Purkinje cells, and cells in the molecular layer (Eftekhari et al., 2013a, b). There is no description of CGRP distribution in the MN of the primate cerebellum (Eftekhari et al., 2013a, b). The difference might be attributed to species, or as suggested by authors, technical reasons (Eftekhari et al., 2013b).

An immunohistochemistry study exploring the fetal development of rats revealed transient expression of CGRP in a subset of inferior olive neurons and established a developmental pattern of CGRP expression in climbing fibers, a subclass of olivocerebellar axons (Chedotal and Sotelo, 1992; Morara et al., 1992, 2001). Specifically, during postnatal development, CGRP-positive climbing fiber terminals were seen to converge onto the cell somas of nearby Purkinje cells (Chedotal and Sotelo, 1992; Morara et al., 1992, 2001; Figure 2). An *in-situ* hybridization study demonstrated that the pattern of olivary CGRP mRNA expression coincided with the spatiotemporal distribution of CGRP immunoreactivity in developing neonatal rats, which provides an explanation for transient CGRP expression (Morara et al., 1995). This developmental pattern suggests that CGRP might play a role in reshaping connectivity and stabilizing synapses in the cerebellar circuitry (Morara et al., 1992, 1995). During development, these CGRP-positive climbing fibers can modulate calcium signaling in proximal astrocytes from neonatal mice, enabling CGRP to exert profound effects on neuronal differentiation (Morara et al., 2008).

Interestingly, studies found the distribution of CGRP throughout mossy fibers to the adult cat cerebellar cortex (Sugimoto et al., 1988; Bishop, 1992, 1995). These CGRP-positive mossy fibers were shown to originate from the brainstem precerebellar nuclei (lateral reticular nucleus, external cuneate nucleus, inferior vestibular nucleus, and basilar pons) in adult cats (Figure 2), suggesting that these structures may function to regulate input into the cerebellar cortex (Bishop, 1992). Based on this finding, Bishop later investigated the physiological role of CGRP in mossy fibers by exogenous application of CGRP to Purkinje cells of adult cats and reported that CGRP both greatly reduced the sensitivity of Purkinje cells to excitatory amino acids and was able to obstruct synaptic activity following stimulation of the inferior cerebellar peduncle (Bishop, 1995). It was also reported that CGRP within cerebellar mossy fibers and serotoninergic neurons had a synergistic effect on inhibiting Purkinje cell firing in response to glutamate (Bishop, 1995). But one caveat is that CGRP is also expressed in the cerebellar cortex and the MN, not exclusively in the mossy fibers, thus exogenous CGRP application cannot completely mimic CGRP release from mossy fibers. However, one study found that CGRP had a protective effect on the homocysteine-induced neurotoxicity in the cerebellar neurons (Abushik et al., 2017). Homocysteine showed an increased level in migraine patients and thus might play a role in migraine (Isobe and Terayama, 2010; Oterino et al., 2010; Abushik et al., 2014). Further characterization of CGRP action on cerebellar neurons is required.

### The expression of CGRP receptors in the cerebellum

The canonical CGRP receptor is a complex of three proteins: receptor activity-modifying protein 1 (RAMP1), calcitonin receptor-like receptor (CLR), and receptor component protein (RCP). Recently, a second CGRP-responsive receptor, amylin subtype 1 receptor (AMY1), which is comprised of RAMP1 and calcitonin receptor (CTR), has also been identified (Hay and Walker, 2017).

Research into the pharmacology of CGRP receptor antagonists revealed that the cerebellum had the most abundant expression of antagonist binding sites (Salvatore et al., 2010; Hostetler et al., 2013). RAMP1 and CLR are reportedly expressed intracellularly in Purkinje cells including their processes, and cells in the molecular layer of the primate cerebellum (Eftekhari et al., 2013b). In the rat cerebellum, RAMP1 was shown to be expressed on the surface of Purkinje cell bodies and their processes, and discovered as fibers in the molecular layer, granular layer, and white matter (Figure 2). RAMP1 was also observed as processes, but not in the cell somas in the MN (Edvinsson et al., 2011, 2020; Warfvinge and Edvinsson, 2019; Warfvinge et al., 2019; Figure 2). CLR immunostaining showed inconsistent results detected by the same group—CLR was found on the surface of Purkinje cell bodies and their processes (Edvinsson et al., 2011). Another study by the same group revealed its expression in somas of Purkinje cells, as fibers in the granular layer, and in both the cell somas and fibers in the MN (Warfvinge and Edvinsson, 2019). There are two possible explanations for the inconsistency; it may be attributed to differences in tissue quality; or high levels of receptor expression rendering the observing immunoactivity to appear intracellular (Eftekhari et al., 2013b). Strikingly, stimulation of afferent climbing fibers originating from the inferior olivary complex induced the expression of CGRP receptors in the anterior lobe of the cerebellum in adult rats (Rosina et al., 1990), suggesting that the cerebellar afferents can regulate the expression level of cerebellar CGRP receptors.

In addition to neurons, Morara and colleagues reported the presence of CGRP receptors in Bergmann glia of the developing (Morara et al., 2000) and adult rat cerebellum (Morara et al., 1998), although the exact CGRP receptor subunit was not clarified. Later, they reported the presence of RCP in Bergmann glia in neonatal mice (Morara et al., 2008). Further, the CGRP receptor showed a developmental expression pattern in the developing rat cerebellum (Morara et al., 2000). During the second postnatal week, CGRP receptors were expressed on the surface of Bergmann glia and cytoplasm of Purkinje cells in the rat cerebellum, while after postnatal day 15, CGRP receptors are expressed on the cell surface of Purkinje cells (Morara et al., 2000). However, a later study found that RAMP1 was not in the adult rat cerebellar glial cells, even though RAMP1 and glial cell marker staining were almost identical (Edvinsson et al., 2011).

In total, CGRP receptors appear throughout different cell types in the cerebellum. Combined with the data demonstrating that the cerebellum contains the highest number of binding sites in the primate brain, it is not surprising CGRP could play role in how the cerebellum processes information. Further studies are needed to determine how CGRP and its receptors contribute to behavioral outputs.

## Animal Studies on The Cerebellum and Migraine

CGRP and its receptor subunits are expressed in the cerebellum, but their contribution toward migraine pathophysiology is unclear. In addition, what other factors contribute to the abnormality of the cerebellum in migraine? Does the abnormality (in volume, activity, and functional connectivity) of the cerebellum play a causal or modulatory role, or is it the consequence of migraine attacks? In this respect, preclinical studies are necessary to resolve these queries. There are many migraine animal models (for more details, refer to Wang et al., 2021). However, to date, few animal studies have been published that investigate the relationship between the cerebellum and migraine. Despite inherent limitations, these models, coupled with brain imaging and molecular approaches, can provide key mechanistic insights into migraine pathophysiology.

### Behavioral studies

Based on the expression of CGRP receptor subunits RAMP1 and CLR in the MN (Warfvinge and Edvinsson, 2019), a recent study investigated the effect of CGRP delivery into the MN on migraine-like behaviors (Wang et al., 2022b). CGRP administration into the MN induced light-aversive behavior in both male and female mice, significant anxiety-like and squinting responses in female mice and more robust tactile hypersensitivity in female mice (Wang et al., 2022b). This discovery suggests that CGRP can act in the cerebellum to induce migraine-like behaviors (Wang et al., 2022b). Later, Wang et al. (2022a) discovered that optical stimulation of CGRP neurons in the MN induced light aversion only in female mice and tactile sensitivity in both sexes. These phenotypes originated from targeting different neuronal populations that are distinct but overlapping. The CGRP administration study targeted CGRP receptor-expressing neurons (Wang et al., 2022b), while the optogenetic study targeted CGRP-expressing neurons (Wang et al., 2022a). It is certainly possible that some CGRP neurons express CGRP receptors, which may result in shared phenotypes. Interestingly, both studies displayed more predominant responses in female mice. Future studies are needed to explore the role of CGRP and CGRP receptors in other cerebellar cells in relation to migraine.

### Imaging studies

Using animal models for brain imaging studies can provide an inherently greater homogeneity of observation than is possible in clinical studies; which can help determine the relationship between a brain region and behavior (Hoyer et al., 2014).

Ictal and interictal phases can be easily achieved by applying triggers to animals. The application of NTG or inflammatory soup to animals has been used to model migraine. In a study conducted by Abad et al., male rats showed increased ^23^Na concentration in the cerebellum ~2 h after NTG treatment (Abad et al., 2018). This suggests an imbalance of sodium in the cerebellum in this NTG-induced acute migraine model (Abad et al., 2018). Moreover, Jia and colleagues used a mouse migraine model induced by the dural application of inflammatory soup at low and high frequency to mimic episodic and chronic migraine, respectively. The fMRI imaging time was chosen 24 h after the last inflammatory soup treatment to mimic the interictal phase, or 1 hour after i.p. injection of NTG to mimic the ictal phase (Jia et al., 2019, 2020). Mice receiving inflammatory soup at low and high frequencies (mimicking the interictal phase) both showed increased cerebellar functional connectivity with the insula (Jia et al., 2020) or anterior cingulate cortex (Jia et al., 2019). Mice receiving inflammatory soup at low frequency and NTG (mimicking ictal phase) also showed an increase in cerebellar functional connectivity with the insula (Jia et al., 2020) or anterior cingulate cortex compared to control mice (Jia et al., 2019). These results support the potential importance of the cerebellum in migraine. It would be interesting to perform imaging such as fMRI to examine the cerebellar activity and functional connectivity in the other migraine models, such as CGRP-induced animal models. These animal models make it feasible to further study molecular mechanisms, including neuronal activities and circuits in the cerebellum.

### Electrophysiological studies

Electrical stimulation of the trigeminal ganglion is another animal model of migraine. Trigeminal stimulation of rats decreased the spontaneous firing rate of Purkinje cells in the acute parafloccular slice both contralaterally and ipsilaterally to the stimulation site (Li et al., 2019). The paraflocculus is a cerebellar lobule and sends inputs to vestibular nuclei (Tabata et al., 2002). Given that Purkinje cells in this region synapse onto vestibular nuclei (Tabata et al., 2002), trigeminal ganglion stimulation-induced inhibition of Purkinje cells might contribute to the occurrence of vestibular migraine (Li et al., 2019).

## Conclusions

Taken together, the combination of: (1) findings that cerebellar connections with pain/migraine-related regions and manipulations alter pain; (2) cerebellar symptoms in migraine patients and; (3) changes in structure, activity, functional connectivity, and metabolism in migraine patients or migraine animal models, all suggest that the cerebellum plays a role in migraine. Based on reports that cerebellar manipulations affect neuronal activities in pain-related brain regions, we propose that the cerebellum might play a modulatory role in migraine. This could happen by cerebellar modulation of the descending pain pathway *via* the brainstem and/or by modulation of the ascending sensory pathway *via* the dorsal column and thalamus. Moreover, direct ascending projections from both the trigeminal ganglia and the SpV to the cerebellum highlight the likely importance of cerebellar modulation in these sensory pathways.

From clinical and preclinical studies described above, the cerebellar regions that most frequently show abnormalities or have been investigated in pain and migraine are Crus I, Crus II, the vermal lobules VI and VIII, lobules IV–VIII, the MN, and the lateral cerebellar nucleus. Future studies dissecting the specific functions of different cerebellar subregions and their circuits will help reveal cerebellar contributions to migraine pathophysiology. Towards this goal, CGRP and its receptors in the cerebellum might be a possible contributor to migraine. Pharmaceutical and optogenetic approaches to modulate CGRP and its receptors may provide new avenues to reveal cerebellar mechanisms and treat migraine pathophysiology.

## Author’s Note

The contents do not represent the views of Veterans Administration or the United States Government.

## Author Contributions

MW, JT, and ND drafted the manuscript. KP, AR, and LS critically revised the manuscript. All authors contributed to the article and approved the submitted version.

## Funding

This work was supported by the National Institutes of Health (R01 NS075599); VA-ORD (RR&D) MERIT (1 I01 RX003523-0); Career Development Award (IK2 RX002010); and Center for Prevention and Treatment of Visual Loss (VA C6810-C).

## Abbreviations

ACC: Anterior cingulate cortex
AMY1: Amylin subtype 1 receptor
CGRP: Calcitonin gene-related peptide
CLR: Calcitonin receptor-like receptor
CTR: Calcitonin receptor
EEG: electroencephalography
FC: Functional connectivity
FHM: Familial hemiplegic migraine
fMRI: functional magnetic resonance imaging
GMV: Gray matter volume
HCs: Healthy controls
i.p.: Intraperitoneal
IS: Inflammatory soup
MN: Medial cerebellar nucleus
NSAIDs: Non-steroidal anti-inflammatory drugs
NTG: Nitroglycerin
PACAP38: Pituitary adenylate cyclase-activating polypeptide-38
PAG: Periaqueductal gray
PBN: Parabrachial nucleus
PET: positron emission tomography
PoT: Posterior thalamus
RAMP1: Receptor activity-modifying protein 1
RCP: Receptor component protein
SpV: Spinal trigeminal nucleus
ZI: Zona incerta.
